# A Roadmap for Ubiquitous Crowdsourced Mobile Sensing-Based Bridge Modal Identification

**DOI:** 10.3390/s25082528

**Published:** 2025-04-17

**Authors:** Liam Cronin, Debarshi Sen, Giulia Marasco, Iman Dabbaghchian, Lorenzo Benedetti, Thomas Matarazzo, Shamim Pakzad

**Affiliations:** 1 Department of Civil and Environmental Engineering, Lehigh University, 117 ATLSS Dr., Bethlehem, PA 18015, USA; gim223@lehigh.edu (G.M.); imd220@lehigh.edu (I.D.); snp208@lehigh.edu (S.P.); 2 Department of Civil Engineering, Southern Illinois University Carbondale, 1263 Lincoln Dr., Carbondale, IL 62901, USA; debarshi.sen@siu.edu; 3 Displaid, Via Carlo Freguglia, 2, 20122 Milano, Italy; lorenzo.benedetti@displaid.it; 4 Department of Mechanical Engineering, United States Military Academy, 606 Thayer Rd., West Point, NY 10996, USA; thomas.matarazzo@westpoint.edu

**Keywords:** mobile sensing, crowdsourcing, system identification, bridge monitoring, ride-share, smartphone

## Abstract

Vibration-based bridge modal identification is a crucial tool in monitoring and managing transportation infrastructure. Traditionally, this entails deploying a fixed array of sensors to measure bridge responses such as accelerations, determine dynamic characteristics, and subsequently infer bridge conditions that will facilitate prognosis and decision-making. However, such a paradigm is not scalable, possesses limited spatial resolution, and typically entails high effort and cost. Recently, mobile sensing-based paradigms have demonstrated promise in laboratory and field settings as an alternative. These methods can leverage big data from crowdsourcing vibration data acquired from smartphone devices belonging to pedestrians and passengers traveling over a bridge, constituting a significantly large data stream of indirectly sensed bridge response. Although the efficacy of such a paradigm has been demonstrated for a limited set of case studies, ubiquitous implementation requires analyzing the impact of vehicle dynamics and quantifying data sources that can be used for the purpose of bridge modal identification. This paper presents a road map for achieving this through dynamically diverse datastreams such as passenger cars, buses, bikes, and scooters. Existing datastreams point towards the implementation of crowdsourced mobile sensing paradigms in urban settings, which would facilitate effective decision-making for enhanced transportation infrastructure resilience.

## 1. Introduction

Structural health monitoring (SHM) is crucial for the maintenance, management, and enhancing the resilience of infrastructure systems. Out of the approximately 617,000 bridges in the United States, 42% are older than 50 years (the design life of a bridge is considered to be 75 years according to AASHTO LRFD specifications), and 7.5% have been deemed to be structurally deficient [1], making SHM a necessity for the effective functioning of the transportation infrastructure network. In the context of bridges, analysis of data from static tests [2,3,4]; images captured by drones [5,6], cameras [7,8,9], or ground penetrating radars [10]; and dynamic structural data recorded through dedicated monitoring networks [11,12] are among the most common methods used in SHM [13]. The first two are typically employed for periodic assessments, whereas dynamic data are often preferred for developing continuous monitoring frameworks. Over the years, several vibration-based damage-detection strategies have been developed, but they mostly focused on using stationary sensing [14]. However, these classes of methods are limited by their scalability, spatial resolution of estimated dynamic characteristics, and associated costs and efforts in deployment and maintenance. A comprehensive review of state-of-the-art bridge monitoring using vibration techniques and applied machine learning can be found in the work of Sun et al. [15].

Recently, mobile sensing technology has gained significant attention in SHM due to its exceptional spatial and temporal resolution, as well as its cost-effectiveness. The goal of mobile sensing is to convert a sparse set of moving measurements of bridge vibrations into their dynamic properties. Many SID methods have been developed to extract bridge dynamics, including frequencies, damping ratios, and mode shapes [16,17,18], from mobile sensing data. Estimating bridge dynamics using mobile sensing has many benefits over traditional stationary sensor networks. First, since the sensors are moving, fewer sensors are needed to achieve a spatial resolution of system identification comparable to a dense stationary sensor network. Furthermore, this also implies that mobile sensors can provide significantly denser spatial resolution compared to their fixed counterparts. Next, the sensors are not dedicated to a single structure. For example, a mobile sensor can sample one bridge, drive down the road, and sample another bridge, lending to its ability to scale to today’s vast transportation infrastructure. Lastly, since the sensors are not physically on the bridge, there are no installation costs, and since they do not experience harsh weather, they do not require regular maintenance. This contrasts with stationary networks that require intensive manual labor and cost to install and maintain the sensing equipment.

Machine learning can have further-reaching applications in mobile sensor bridge monitoring. The application of machine learning techniques for drive-by approaches is still relatively unexplored; the authors follow by reporting some of the most interesting studies. Cerda et al. [19] proposed a Support Vector Machine (SVM) trained to classify damage typology from a set of frequencies obtained from drive-by measurements. Malekjafarian et al. [20] trained an Artificial Neural Network (ANN) using the responses from multiple runs on undamaged bridges. The trained network can predict vehicle response and frequency content, and a damage index is proposed based on the difference between predictions and observations to detect damage. This work has been extended by Corbally and Malekjafarian [21] to consider the effect of temperature and vehicle speed, demonstrating the approach’s capability to recognize changes in bridge boundary conditions, cracks in the deck, and simultaneous damages. Hajializadeh et al. proposed two studies based on CNN-based damage detection algorithms using mobile sensor data. The first study [22] employs a numerical model of train–track–ballast–bridge interaction and trains the CNN with the continuous wavelet transform (CWT) map of the measured acceleration response during each train passage. The second study [23] extends this work to real data, using images of time–frequency spectrograms as the input. Liu et al. [24] introduced a semi-supervised approach utilizing autoencoders with nonlinear functions for damage localization and severity estimation, leveraging responses obtained from passing vehicles. The results of the study, which were subsequently improved by integrating physics information to guide the algorithm [25], showed a nonlinear trend in vehicle responses with varying damage severity.

Recent work has expanded mobile sensing to include crowdsourcing datastreams with varying success in estimating frequencies and mode shapes [26,27,28,29,30], focusing on data collected from passenger vehicles. The central idea is to acquire large datastreams of rideshare data from drivers crossing bridges in their daily lives to estimate the bride dynamics from the vehicle’s vibrations. Cronin et al. [26] analyzed Uber data and developed a crowdsourcing application to automate data collection on bridges, and implemented a system identification methodology in four real-world case studies. Current work has been focused on data collected from passenger vehicles. However, ride-share companies have expanded to include micro-mobility forms of transportation: e-scooters and e-bikes. Information and Communication Technologies (ICT) are characterized by large volumes of passively generated big data [31]. The growing popularity of micro-mobility data has also led to the development of open crowdsensing platforms [32] for research purposes. Within this context, mobile sensing technology holds significant potential for a wide range of applications, with Structural Health Monitoring (SHM) being one of the most promising [33].

Despite the extent of the datasets being limited with respect to potential global implementation, the authors of [26] show a large-scale proof of concept for implementation and analysis in a field setting. In a crowdsensing application of mobile sensing, there are additional variables to be considered compared to controlled laboratory experiments: (1) the unknown road profile adds noise to the collected measurements, (2) bridge vibration response measurements are acquired indirectly from passing vehicles, which have their unknown dynamics that can obfuscate bridge dynamics, and (3) there is additional noise and uncertainties stemming from the sensor quality and placement inside the vehicle. Addressing these critical challenges is crucial to the ubiquitous use of crowdsourced mobile sensing for bridge modal identification and monitoring.

Many approaches have been developed to avoid or mitigate the impact of vehicle dynamics in mobile sensing. On the data collection side, vehicles have been specially designed so that the vehicle dynamics are known and ideally non-overlapping with the bridge frequencies of interest. For example, the authors of [34] developed a single-axle rigid trailer (no suspension and solid tires) to remove the vehicle component. An alternative approach to remove or undo the effects of the vehicle is to estimate the contact point force, i.e., estimate the input to the system in the post-processing of the data. The authors of [35] adopted this approach for an undamped single-degree-of-freedom system, eliminating the complexity of real suspension systems where damping and nonlinearities can play a key role. The authors of [36] experimentally tested the direct solution on a single axle trailer; these experiments demonstrated improved modal identification around the vehicle frequency at higher speeds. The authors of [37] proposed a stochastic method for multi-degree-of-freedom systems with known damping using a Gaussian process latent force model for vehicle contact point estimation and bridge monitoring. The proposed methodology was tested numerically and again showed the benefits of using the contact point to recover higher bridge modes. The authors of [38] proposed a machine-learning method to estimate the contact point in real vehicles. The neural network was trained on input–output acceleration data at the tire level and in the cabin, which inherently captures the nonlinearity observed in real suspension systems. This study showed very promising results but was untested for bridge-monitoring purposes. Although the last two methods account for more complex systems, their widespread use is limited by assumptions made about the model, the need for prior knowledge of the dynamic properties of the vehicles, or unscalable data collection practices.

Expanding mobile sensing to various forms of transportation creates new technical challenges when indirectly measuring bridge vibrations. For example, passenger car suspensions vary from car to car, and even more dramatic variations in dynamics occur between the vehicle classes, such as passenger cars versus buses, bikes, and scooters. Consequently, estimating the contact point becomes difficult. This work surveys the dynamics of these different modes of transportation and shows the impact of mechanical property variations on existing mobile sensing-based system identification methods. In this work, the scooter data from the ride-share company Superpedestrian are first quantified in Seattle. This is followed by a survey of vehicle dynamics for various buses, scooters, and passenger vehicles. Next, numerical results are presented to discuss the effect of varying vehicle dynamics on the modal identification process. This simulation discusses both variability in dynamics for passenger vehicles and drastically different systems, such as between scooters and vehicles. Last, there is a discussion on the results and guidance toward new research areas, along with a discussion of how to leverage this diverse set of vehicles to benefit analyses.

## 2. Methods and Results

### 2.1. Vehicle Dynamic Survey

**Figure 1:**
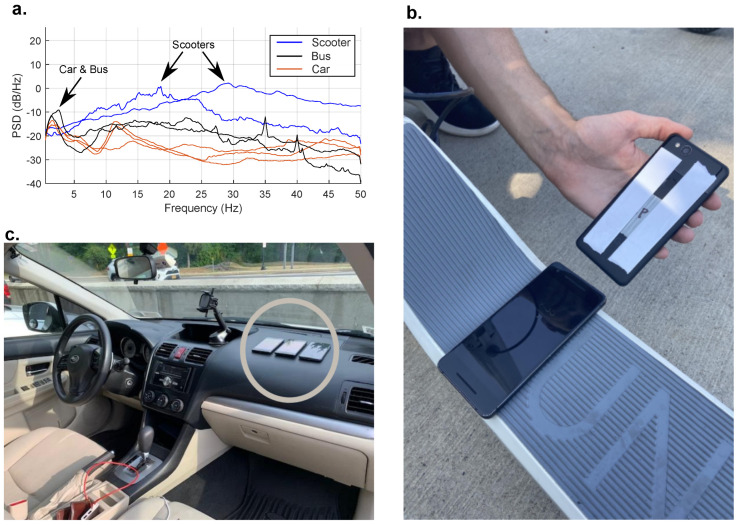
Survey of vehicle dynamics for scooters, busses, and passenger vehicles. (**a**) PSD of operational acceleration measurements from smartphone on board different modes of transportation. (**b**) Photograph of sensor placement on scooters, secured using double-sided tape on the foot platform. (**c**) Photograph of the sensor placement in the passenger vehicles with a set of phones secured on the dashboard with double-sided tape.

Part of the challenge of using the proposed datastreams for indirect bridge monitoring stems from the fact that mobile sensors have widely varying dynamics. Car suspension properties vary depending on the manufacturer/model and are dynamically very different from other classes of vehicles such as scooters or bikes. As a result, the signals collected from sensors deployed inside a vehicle can be significantly altered with respect to the input to the system and relatively from vehicle to vehicle. To elucidate the extent and impact of the differing systems, operational acceleration responses were collected, and the PSDs of the measurements from cars, buses, and scooters can be seen in Figure 1.

In this survey of vehicle dynamics, acceleration measurements were collected from within various passenger cars (dashboard mounted), atop scooters (foot platform), on bikes (frame mounted), and in buses (floor). The acceleration response was measured using smartphones during regular operation using the Phyphox app [39]. Passenger vehicle measurements were recorded while driving in traffic, similar to bus measurements as a passenger and a phone secured to a hard surface. The e-scooter and bike measurements were recorded on a pedestrian path while traveling at a variety of speeds and different locations on the pavement. The types of vehicles used can be seen in Table 1.

Assuming the road profile has relatively flat spectral characteristics, the peaks in the spectra would be primarily from the vehicle’s transfer functions. In Figure 1a, these peaks are annotated. Generally, passenger vehicles’ first mode occurs around 1–3 Hz, and their second occurs around 10–15 Hz, which aligns with this survey of passenger vehicles. The bus measurements show peaks in the same range as the passenger vehicles.

In stark contrast, the scooters are significantly more rigid structures without higher-quality air-filled tires or suspension systems. Not all scooters are alike, and personal e-scooters might have these features for a more comfortable ride rather than low maintenance costs. These scooters are publicly available on ride-share platforms. This study collected data on two scooters: Bird (ride share) and Unagi (online rental). In the collected operational data, the spectra in Figure 1 show a single peak for both scooter tests with peaks around 20 Hz and 30 Hz. These results demonstrate the significant variations one may encounter when deploying a crowdsourced mobile sensing paradigm. In the subsequent section, the effect of such variations on the performance of modal identification algorithms used for mobile sensing is discussed.

### 2.2. Numerical Study

The mobile sensing numerical analysis used a simplified simulation approach, where the bridge and vehicle are modeled separately instead of a fully coupled vehicle bridge interaction simulation without significant loss of accuracy [40]. In this approach, the loading on the bridge due to the environment and traffic is approximated to be Gaussian white noise, and the interaction between an individual vehicle and the bridge is assumed to be negligible. These assumptions hold for long-span bridges with a small vehicle-to-bridge-mass ratio only. To generate data following this procedure, a finite element bridge model is loaded at the nodes with Gaussian white noise. This sample bridge is a unit length consisting of 500 Euler–Bernoulli beam elements with the mass and stiffness properties chosen such that the first five frequencies of the bridge are 0.15, 0.63, 1.41, 2.51, and 3.93 Hz. The damping ratio was chosen to be 1% for all modes. The goal of choosing these properties was to have bridge frequencies lower than and overlapping the vehicle frequencies. The displacement response is calculated for a set time frame. Then, mobile trips are extracted by interpolating across the spatiotemporal response of the bridge where a vehicle crosses. Finally, this is used as an input to a car model, and the response within the cabin of the vehicle is estimated.

In order to focus only on the effect of vehicle dynamics, the vehicle paths, velocities, and orientations are not varied. However, it should be noted that the system identification method can accommodate random vehicle paths and varying speeds. The bridge is described as unit length, and the vehicle speeds are described in terms of the time to cross and temporal spacing. The underlying parameter for this simulation is the number of samples each sensor takes as it traverses the bridge. In this simulation, the vehicles take 60 s to traverse the bridge, while traveling at a constant speed, and are spaced two seconds apart (meaning every two seconds, a car enters the bridge). The acceleration measurements from the bridge at the location of the vehicle are then input into the quarter-car model (the assumed vehicle model), and the sprung mass (representing the inside of a vehicle cabin) acceleration measurements are recorded.

**Figure 2:**
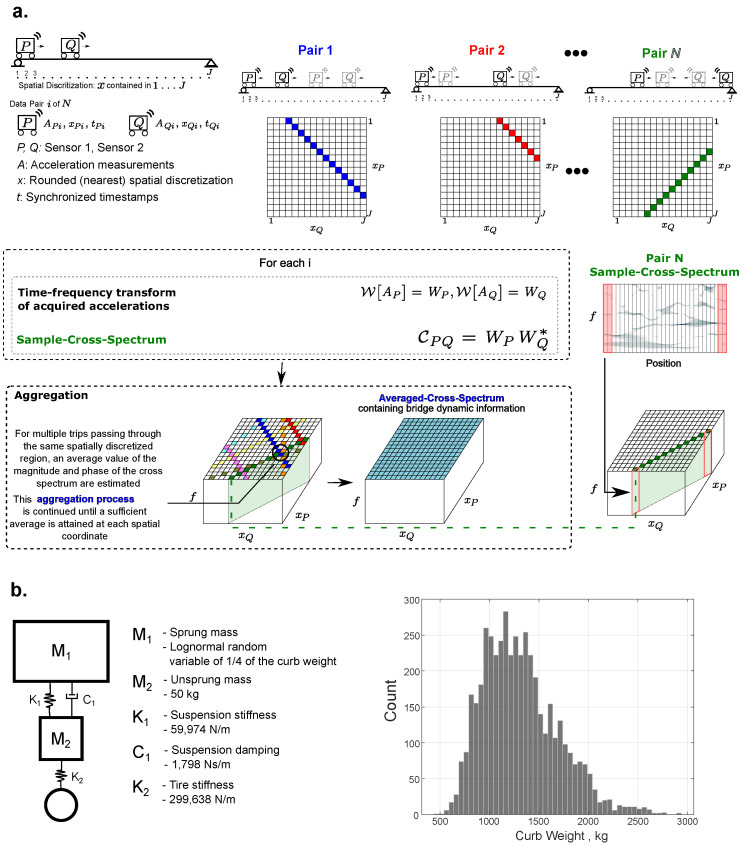
(**a**) Summary of aggregation methodology presented in [16] (**b**) The quarter-car model used for modeling vehicle dynamics with the sprung mass modeled as a random variable of 1/4 of the curb weight. These discrete values used for the remainder of the properties are from a quarter-car model used in a numerical study by the authors of [42].

The system identification method used here spatially averages the time–frequency cross-spectra calculated from the acceleration measurements of moving sensors and the positions of the sensors at each time instant [16]. Figure 2a summarizes the methodology. For this method, two sensors must be on the bridge simultaneously. First, a time–frequency transform, the Short-Time Fourier Transform (STFT), is applied to the sensor acceleration signals, and time–frequency cross-spectra is calculated between the sensor pair. This is calculated following Equation (1). The cross-spectra from each pair of signals are averaged with respect to the locations of the sensors at each time instant.

(1) $$W\left[A_{P_{i}}\right]=W_{P_{i}},\;W\left[A_{Q_{i}}\right]=W_{Q_{i}},\;C_{PQ}=W_{P_{i}}W_{Q_{i}}^{*}$$

The spatial aggregation requires the bridge to be segmented, which controls the spatial resolution of the SID. This is one of the user-defined parameters of the method. The goal is to spatially aggregate the frequency contents with respect to the positions of the sensors, which can be most easily visualized by plotting the position of one sensor versus the position of the other sensor. A straightforward implementation of the method is a running average, meaning that, given the sensor path, the frequency contents are added to the nearest global coordinate (segment) and rescaled per the count. The complex average in Equation (2) is calculated at each spatial coordinate. In this simulation, all results use 1500 sensor pairs aggregated to 19 evenly spaced global coordinates (5%,10%...95% of the bridge length). Once the frequency response matrix is estimated, Frequency Domain Decomposition is used to determine the modal properties.

(2) M^avg=1N∑i=1NM^i=1N∑i=1NRe[Mi]^+i1N∑i=1NIm[Mi]^

This simulation uses a quarter-car model to mimic the vehicle suspension, and the sprung mass ($M_{1}$) is modeled as a lognormal random variable, as shown in Figure 2b. To approximate a distribution for the sprung mass, a database of vehicle specs was acquired from an online database [41]. In this database, 4637 vehicles were listed with the make, model, class (i.e., hatchback, station wagon, sedan, etc.), and curb weight (kg). The curb weight, i.e., the total weight of the car, was used to approximate the sprung mass since the actual value of the sprung mass is unknown. The distribution of weights is shown in Figure 2b, and in the simulation, this was modeled as a log-normal distribution to avoid negative valued weights. This database does supply some general information on the type of suspension. However, the specifics relevant to modeling the dynamics are unknown. This is common across online information regarding suspension properties. For this reason, only the sprung mass was assumed to be a random variable, and all other parameters (stiffness and damping ratio of the suspension components) were assumed to be a constant. The authors believe this is sufficient for discussing the effects of mobile sensing system identification, even if it does not perfectly mimic reality.

**Figure 3:**
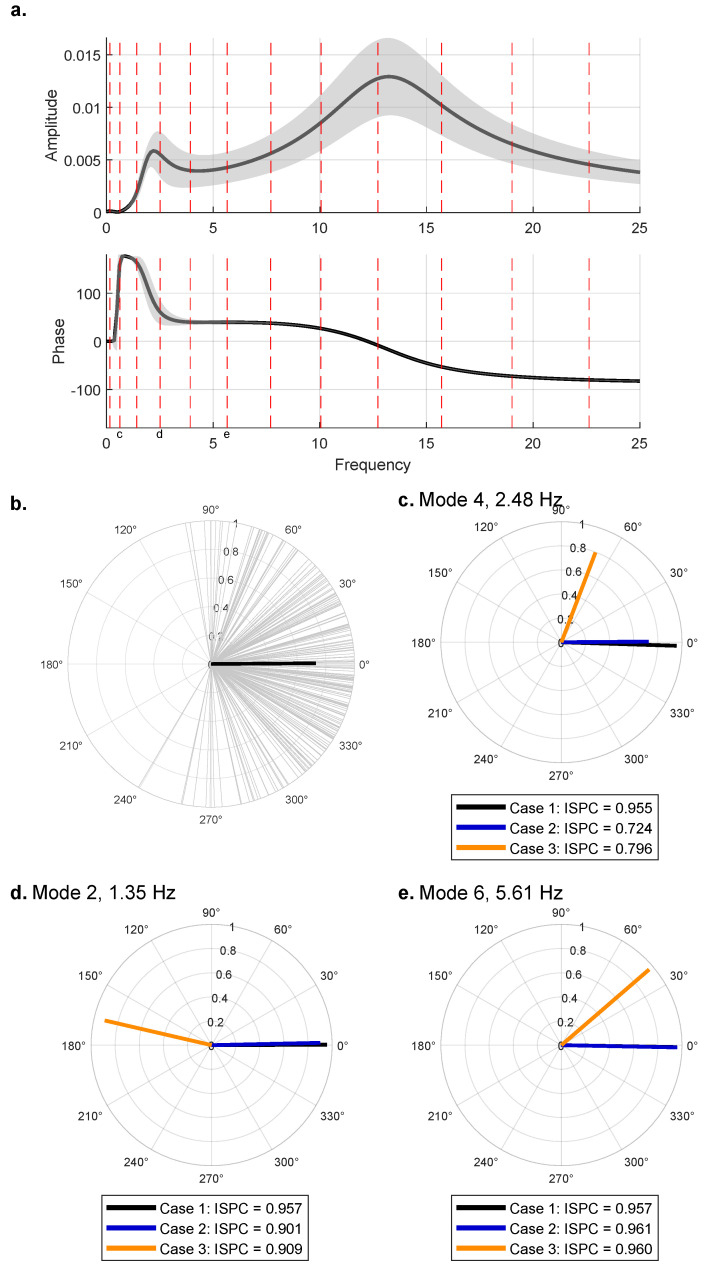
(**a**) The frequency response function of the quarter-car model shows a distribution (2 standard deviations) for the variability of the sprung mass in light gray and bridge modes notated as dotted red lines, (**b**) a polar scatter of the phase differences at 2.48 Hz in the values used in the aggregation process with the dark gray as the ISPC in Case 2 subplot C. (**c**–**e**) Polar graphs of the ISPC for three cases of mobile sensor aggregation at three different bridge modes.

Figure 3a shows the frequency response function of the quarter car with a shaded distribution to show the effect of the variation of the sprung mass $M_{1}$. For this analysis, the phase change due to the suspension is critical, and around the first modal frequency, it can be seen that there is high variability. When estimating the response matrix in the simulation, the frequency contents from each sensor pair used in the averaging process were saved. Figure 3b shows the *phase difference* from each trip plotted as light gray unit vectors. The dark black line shows the mean direction of the samples in the distribution, and the length of the vector is the Inter-Site Phase Clustering (ISPC) value, which is a distribution metric of the phase angle with a maximum value of one, calculated using Equation (3). ISPC is calculated by averaging unit vectors with phase angles from the phase differences in the vehicle trip signals. The magnitude of this is the ISPC, meaning if all of the phase angles in the average were the same, the result would be 1, and increasing the variability would result in a value closer to zero. These values are taken at the fourth mode of the bridge, 2.48 Hz. Figure 3c plots three cases at this mode of the bridge. Case 1 is the measurements taken directly from the bridge (no quarter-car model). Case 2 is with the quarter-car model with $M_{1}$ as a random variable. Last, Case 3 is a pair of trips where one has the quarter car and the other is direct bridge vibrations. Figure 3d,e shows this result at two other bridge modes.

(3) $$ISPC=\left|\frac{1}{N}\sum_{n=1}^{N}e^{i\Delta \phi_{n}}\right|$$

Across all frequencies, the direct bridge vibrations, Case 1, have an ISPC of 0.95, which can be used as a reference for the ideal case. The distribution when the vehicles are involved has much higher variability depending on the frequency of interest. Mode 4 has the highest variability as it aligns with the first mode of the vehicle, as noted in Figure 3a. Even though the variability is high, the mean still aligns with the reference case of the direct bridge vibrations case. This is true at all frequencies for Case 2. Case 3, where one sensor in the pair includes the quarter-car model and the other is direct bridge vibrations, shows a potential problem of aggregating data from mobile sensors with drastically different suspension systems. Case 3 essentially shows that the vehicles crossing the bridge oscillate out of phase at the second modal frequency of the bridge, which affects the ability to use these signals for system identification purposes. If the direct bridge vibration case resembles an e-scooter, which is very rigid, and is aggregated with regular passenger vehicles, the mean of the distribution can be skewed, as seen in Case 3.

Even though aggregating data from mobile sensors with widely varying vehicle dynamics can skew results, they can still be used concurrently. The easiest solution is to aggregate similar vehicles together so that if the system’s transfer function significantly alters the input signal, all vehicles in the aggregation have the same relative changes (plus variability). This is what happens in Case 2 in this analysis. The vehicles alter the input signal. However, since all vehicles have the same general transfer functions, the aggregation still has a mean around the true values because the relative difference between vehicles is small. Subsequently, the different classes can be aggregated.

### 2.3. Quantifying e-Scooter Usage for Bridge Monitoring

Crowdsourced mobile sensing has focused on leveraging datastreams from passenger cars. Currently, there exists no framework to collect such data from the general public, except for access provided by ride-share companies. Those same companies have expanded their products to offer e-scooters and bikes in many cities. With public bus transit and regular passenger car usage, many avenues exist for data collection on any given bridge in an urban setting. This paper focuses on Seattle, Washington, as a case study to quantify and discuss the extent of available data. To achieve this, local and federal governments’ traffic and pedestrian count databases and a dataset supplied by Superpedestrian, a ride-share company, are used. The Superpedestrian database consists of approximately 105,000 entries of the origin–destination of scooter trips around the city of Seattle within a year (i.e., tabulated data of the ride start time, date, and GPS coordinates of the start and end of the route). The available dataset is significantly larger than traditional sources of SHM data; however, compared to market competitors, such as Uber, Lyft, or Lime, Superpedestrian has a substantially smaller market share and, since 2023, has ended US operations.

With origin–destination data, the exact route is unknown; only the beginning and end locations are recorded. To estimate the crossed bridges for each trip, an assumption was that the rider took the shortest route. In conjunction with OpenStreetMap, an open-source map with a user API, the bridges along the shortest path are logged. Repeating this estimation process for all trips, a log of approximately 275,000 bridge crossings was discovered. The number of bridge crossings is higher than the number of trips because one rider can cross more than one bridge for a trip, and there are many bridges within the city of Seattle. Furthermore, within OpenStreetMap, many objects are considered bridges, like overpasses, which are included in the 275,000 crossing count.

**Figure 4:**
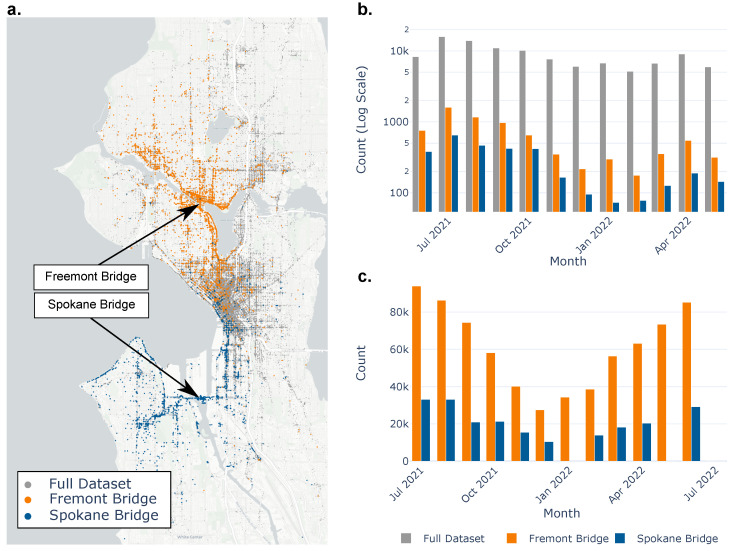
(**a**) Geoscatter of the origin of Superpedestrian scooter trips with the full dataset plotted in grey and trips that included the Freemont and Spokane bridges highlighted in orange and blue. The figure was created using the python library Plotly (version 6.0.1) with Mapbox integration [43,44]. (**b**) Bar chart of the scooter usage over the year of data collection, and bridge crossing for the two case bridges. (**c**) Bar chart of the pedestrian path usage from a survey provided by the local government [45].

Two specific bridges are analyzed in more detail: the Freemont Bridge and the South Spokane Bridge. Figure 4a shows a map of Seattle with the start locations of the scooter trips shown in grey. The rides estimated to have crossed the Freemont Bridge are highlighted in orange, and the rides that crossed the South Spokane Bridge are in blue. Qualitatively, the concept of micro-mobility, i.e., movements of pedestrians within a city, can be observed. The Fremont Bridge, located on the northern side of the city, is surrounded by orange scatter points, and those points are localized to that region of the city. This means that riders who crossed the Fremont Bridge only traveled short distances; they would start their scooter trip just south of the bridge or just north of the bridge. This same concept is also seen around the Spokane Bridge and the blue scatter of the trips that crossed the bridge.

Figure 4b shows how the total trips and scooter crossings on the two case bridges vary throughout the year. Within this dataset, total rides varied between about 8000 and 10,000 trips per month. Scooter trips, as to be expected, fluctuate depending on the time of the year. This trend can be seen in the aggregate and on the individual counts for each bridge case. In warmer months, there are significantly more trips than in winter months, with a peak in ride usage in the summer of 2022. Figure 4c is a local government-funded, multi-year pedestrian path usage study [45]. Here, the same time frame as the scooter dataset is presented for each case bridge. The Figure 4b scooter trips are a subset of the Figure 4c total pedestrian path usage and the total usage also follows the same trends shown in the scooter data.

The average annual daily traffic of the Freemont Bridge (240 ft span, steel truss bridge) and SW Spokane Bridge (480 ft span, concrete swing bridge) are 29,300 and 11,000, respectively [46]. In contrast to passenger vehicles or public bus transit, which would not have as significant seasonal trends, varying usage here may have further implications regarding the ability of scooters or bikes to provide a sufficient datastream year-round for continuous monitoring. Considering that Superpedestrian only had a small market share in 2021–2022 (subsequently left US operations), only 50 to 100 scooter crossings occurred over the entire month of January on the Spokane bridge. This number of trips is at the lower limit shown in real-world case studies for finding frequencies and the absolute, and even though a MAC value greater than 0.90 could be estimated, according to the analysis of sample size vs. accuracy, higher accuracy estimates should be possible with more data [26]. Assuming movement patterns of scooter trips are similar across other ride-share companies and that Superpedestrian only makes up a small percentage of ride-share usage in Seattle, a significantly larger dataset should be available and possibly continuous mobile sensor SID.

## 3. Discussion

Crowdsourced, field-tested implementations of mobile sensing methods are not common for many reasons. First and foremost, there is an inherent difficulty in data collection for research groups, as the topic premise is crowdsourcing from ride-share/government agencies. Most, but not all, works use data collected by the research group using smartphones as a proxy for analysis, and those that use actual crowdsourced data are limited in the quantity of data and accompanying metadata for further analysis. This work highlights an issue applying system identification methods that rely on synchronous sensors measured from vehicles that differ dynamically. To investigate the variability of vehicle dynamics, spectra from operational measurements from various modes of transportation show how natural frequencies vary. The first mode from the passenger cars and busses was similar (1–3 Hz); however, the scooters had a significantly higher first mode (20–30 Hz). In the initial numerical study, it was shown that the effects can be minimized if dynamically similar vehicles are aggregated first and then subsequently aggregated by different modes of transportation. This means that in a full-scale experiment, it is advised that these modes of transportation be aggregated separately.

Even though having a diverse dataset adds uncertainty to the estimation process, drastically differing dynamics pose an opportunity to uncover additional information from the bridge. The vehicle’s natural frequencies are critical parameters concerning mobile sensing system identification because the bridge dynamics will be challenging to identify around them. This effect has been explored with previous studies showing the vehicle dynamics overshadowing the bridge vibrations. The previous works focus on using a single sensor with a single set of dynamic properties, and on overcoming this, studies proposed algorithms to estimate the input to the vehicle or specially designed trailers with known dynamics. For example, the authors of [34] developed trailers with high natural frequencies that do not overlap with bridge modes of interest. A key takeaway from this survey is that the datastreams have natural frequencies in distinct ranges. There is potential to have these datastreams monitor different frequency ranges. For example, passenger cars may have difficulty identifying 1–3 Hz bridge modes. However, scooters do not. Rather than trying to estimate the input and undo the effect of the vehicles, the goal would be to use different mobile sensors to monitor the obscured frequency range. Furthermore, e-scooters carry many of the same benefits of a rigid mobile sensor as the trailers discussed in [34], but with the added benefit that they are already deployed around the world in major cities. With further investigation, optimized aggregation methods can be developed to leverage subsets of the available data to improve estimation accuracy.

Recently, Dabbaghchian et al. [47] used machine-learning methods for a similar sorting task on raw signals to find more informative subsets to the full crowdsourced dataset. The study employs a convolutional neural network (CNN) to predict a relative quality score for each trip. This prediction is based on a feature vector that incorporates both frequency and spatial information derived from the trip’s acceleration signal. The work also showed that if there are not enough data, the result is suboptimal. On the other hand, if all the data are aggregated, including the poor signals, the results are also suboptimal. Instead, a most informative subset of the data exists. The paper was based on a methodology using asynchronous mobile sensing data and absolute mode shape identification, meaning the effect of the vehicle discussed in this work was not incorporated in the analysis. However, an aggregation methodology using metadata or signal characteristics would be highly beneficial. This allows one to optimize which sensor pairs aggregate to minimize the detrimental effects of varying vehicle dynamics. Moreover, assessing signal quality requires a reference, typically obtained from fixed sensor deployment campaigns, which can be expensive. This necessity often leads to the use of supervised machine learning. However, unsupervised machine-learning methods may offer a more cost-effective and advantageous alternative and that requires novel ideas for the quality analysis of the data collection.

Recent work showed bridge dynamics can be extracted from crowdsourced data from ride-sharing platforms [26,27]. These pilot studies only scratch the surface, showing the information exists in the dataset and can be extracted using signal-processing techniques. Orders of magnitude larger datasets could be accumulated rapidly from multiple datastreams from public bus transportation to ride-share cars and scooters.

## 4. Conclusions

This work focuses on expanding mobile sensing for bridge monitoring to include additional datastreams of buses, e-scooters, and passenger vehicles. Previous works looked into quantifying passenger vehicles for mobile sensing. Here, e-scooter origin–destination data from the ride-share company Superpedestrian was used to quantify scooter trips on bridges and showed that e-scooter usage, along with bike path usage, was seasonal, with significantly fewer trips in winter months. Next, a survey of vehicle dynamics was conducted by collecting operational acceleration data from these forms of transit. The busses tested showed a fundamental frequency comparable to passenger cars (1–3 Hz), while the scooters had a significantly higher frequency (20–30 Hz). Last, a simulation was performed to analyze the effect of widely varying vehicle dynamics for indirect bridge monitoring with the following takeaways:

- E-scooters have the potential to be good mobile sensors with their rigid dynamic characteristics and are relatively slow-moving compared to passenger vehicles, and the data in this study show potential to collect a significant dataset over inner-city bridges.
- Collecting data from a wide range of classes of vehicles with drastically different dynamics could solve the problems of identifying bridge modes around vehicle frequencies.
- Variability in vehicle transfer functions around modal frequencies adds to uncertainty in the aggregation process due to the varying phase differences between mobile sensor pairs.
- The relative transfer function between mobile sensors is critical, and only the same class of mobile sensor should be aggregated.

## Figures and Tables

**Table 1:** Survey of vehicles and models.

Category	Models
Passenger Vehicles	2012 Subaru Imprezza
	2014 Chevrolet Cruze
	2012 Chevrolet Cruze
E-Scooters	Bird
	Unagi
Busses	Lehigh Campus Bus
	SIU Campus Bus

## Data Availability

Superpedristrian data used in Section 2.3 will not be available, but code/data used elsewhere in this paper will be available upon request.
